# Using Social Media To Increase HIV Testing Among Men Who Have Sex with Men — Beijing, China, 2013–2017

**DOI:** 10.15585/mmwr.mm6821a3

**Published:** 2019-05-31

**Authors:** Liming Wang, Dylan Podson, Zihuang Chen, Hongyan Lu, Vania Wang, Colin Shepard, John K. Williams, Guodong Mi

**Affiliations:** ^1^Division of Global HIV and TB, CDC China, Beijing, China; ^2^Public Health Institute Global Health Fellowship, Washington, D.C.; ^3^Blued.com, Beijing, China; ^4^CDC China; Beijing, China; ^5^Department of Geography, University of California, Santa Barbara, California; ^6^Division of Global HIV and TB, Center for Global Health, CDC; ^7^National Center for AIDS/STD Control and Prevention, CDC China, Beijing, China.

The prevalence of human immunodeficiency virus (HIV) infection in China is low overall (0.06%) (*1*); however, it is substantially higher (8.0%) among men who have sex with men (MSM) (*2*), and the stigmatization of same-sex behaviors in China presents challenges for HIV prevention and treatment efforts. In 2015, Blued, a Beijing-based media company that operates an online dating application popular among Chinese MSM, launched an ongoing HIV testing campaign that combined its push-notification^†^ platform and geolocation capabilities to encourage HIV testing among MSM in Beijing. To assess trends in use of HIV testing services, Blued and CDC’s China HIV program examined testing at six Blued-operated Beijing HIV testing centers from 2 years before the campaign launch in 2015 through December 31, 2017. A sharp increase in HIV testing followed the launch of Blued’s online campaign, indicating that leveraging social media platforms and their geolocation-based text messaging functionality might be useful in increasing HIV testing among MSM, particularly those aged ≤35 years.

Cross-sectional studies in China suggest that MSM have a higher prevalence of HIV infection (*3*). Data indicated that the prevalence of HIV infection among MSM in China increased from 1.0% in 2003 (*1*) to 8.0% in 2015 (*2*). MSM population size estimates in China range from 5 million to 10 million; 50%–75% of HIV-positive MSM are unaware of their HIV status (*1*,*4*).

Effective high-yield testing is an entry point for HIV care and treatment (*5*); because China has >660 million smart phone users (*6*), mobile applications might be effective in targeting MSM. Blued, a Beijing-based media company that operates the largest gay male–oriented social media and geosocial networking mobile application in China, was launched in 2012, and as of 2016, had approximately 27 million registered users and 12 million monthly users in China. Since 2013, Blued has operated six drop-in testing sites in Beijing designed to provide HIV testing in a gay-friendly environment; these six sites served approximately 700 MSM per month in 2017.

In 2015, Blued launched an online campaign to promote HIV testing at its drop-in sites. Using the application’s GPS-tracking capabilities, the campaign began with a one-time mass message push through the application’s private message functionality in March 2015, encouraging users to get tested for HIV while they were within Beijing municipality. The campaign’s outreach efforts were carried out within the framework of Blued’s service agreement with its users, and HIV testing at Blued’s drop-in sites conformed to local and national regulations. After the first message push, the campaign continued with monthly electronic banner promotions of HIV testing on the application’s launch screen. In July 2016, an online HIV testing appointment platform was embedded in the application, which made the online HIV testing promotion routinely available through Blued. Users who accessed the links in the advertisements were redirected to a cellular phone number–based online appointment system, through which they could schedule testing at a nearby testing site. After scheduling the appointment, the selected site and date were sent to the user’s cellular phone to confirm the appointment via text message. At the testing site, after providing written informed consent, participants were asked to provide basic demographic information (Blued nickname, birth date, telephone number, and college student status) and any HIV testing history and results. To adhere to the national mandate for anonymous HIV testing, names and national identity numbers were not collected. Only screening tests were recorded at the drop-in testing sites, but all persons with positive rapid test results were contacted and referred to receive confirmatory testing through local health authorities.

To assess the impact of the social media–based HIV testing promotion campaign, CDC’s China HIV program (supported by the U.S. President’s Emergency Plan for AIDS Relief [PEPFAR]) helped Blued conduct a secondary analysis of the data collected during 2013–2017 from the six Blued drop-in sites. Programmatic testing data were deduplicated to achieve a person-based analytic data set using a unique identification number created with participants’ reported date of birth and telephone number. Blued owned the raw data and created the deidentified analytic dataset to allow secondary analyses. CDC China led the analysis and the report development. Trends in the number of HIV tests, the characteristics of persons tested, and factors associated with receiving a positive HIV rapid test result were analyzed using bivariate and multivariate logistic regression analyses. Analyses were performed using SAS software (version 9.3; SAS Institute) and p-values <0.05 were considered statistically significant. The protocol was reviewed and approved by the institutional review board of the National Center for AIDS/STD Control and Prevention of CDC China (IRB00002276).

After the campaign’s launch in March 2015, HIV testing volume increased sharply: 145 HIV tests were reported during March (Figure 1), a 77% increase over the 82 tests reported during January and February. The total number of tests in 2015 (3,363) represented a greater than threefold increase over the 836 tests received during 2013 and nearly a sevenfold increase over the 425 tests received during 2014 (Figure 2). The number of tests continued to increase to 6,330 in 2016 and 7,315 in 2017, representing 10 times (2016) and 12 times (2017) the average number of annual tests received during 2013–2014.

**Figure 1:**
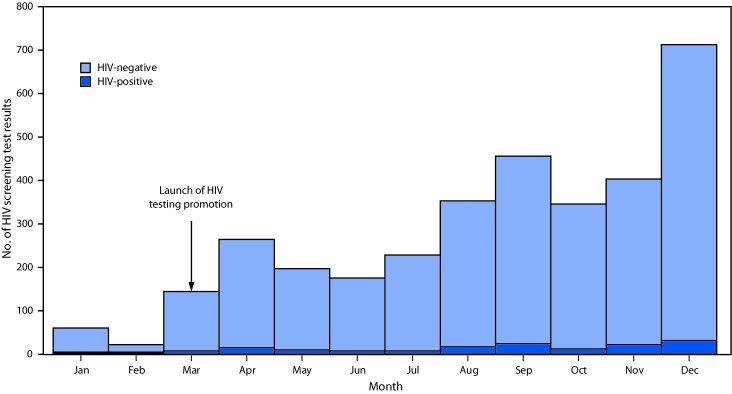
Number of negative and positive human immunodeficiency virus (HIV) screening test results, before and after the HIV-testing promotion campaign launch at six drop-in sites supported by Blued,* by month — Beijing, China, 2015 * A Beijing-based media company that operates an online dating application popular among Chinese men who have sex with men.

**Figure 2:**
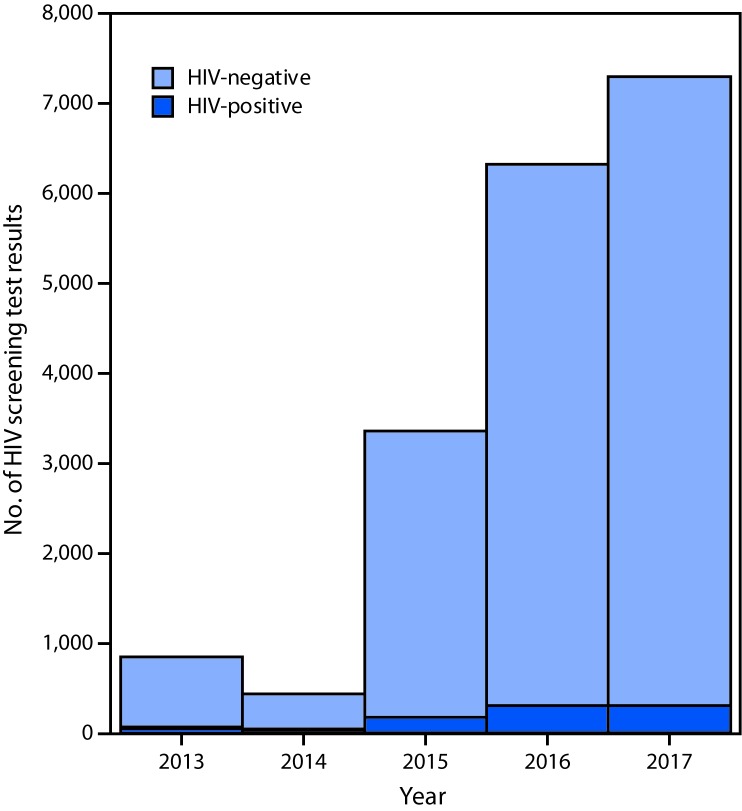
Number of negative and positive human immunodeficiency virus (HIV) screening test results at six drop-in sites supported by Blued,* by year — Beijing, China, 2013–2017 * A Beijing-based media company that operates an online dating application popular among Chinese men who have sex with men.

Overall, 15,932 MSM had 17,008 cumulative HIV test results recorded at one of the six Blued drop-in sites during 2015–2017. Among these MSM, 14,050 (88.2%) were aged ≤35 years (median age = 27 years [interquartile range = 24–31 years]), and 2,693 (16.9%) were college students (Table). Nearly half of participants (44.8%) had never had an HIV test. Sixty-eight percent of participants scheduled their HIV test through the link embedded in the campaign’s private messages and advertisements using Blued.

**TABLE:** Number and percentage of men who have sex with men (N = 15,932) who tested positive for human immunodeficiency virus (HIV) at drop-in sites supported by Blued,* by selected characteristics — Beijing, China, 2013–2017

Characteristic (no. tested	HIV-positive no. (%)	HIV-negative no. (%)	Chi-square test p-value	HIV-positive			
				Bivariate analysis		Multivariable analysis	
				OR (95% CI)	p-value	aOR (95% CI)	p-value
**Age (yrs); median age = 27 years, IQR = 24–31**							
≤35 (14,050)	592 (4.2)	13,458 (95.8)	<0.001	1.0 (—)	<0.001	1.0 (—)	<0.001^†^
>35 (1,882)	131 (7.0)	1,751 (93.0)		1.70 (1.40–2.07)		1.54 (1.26–1.88)	
**College student**							
No (13,239)	664 (5.0)	12,575 (95.0)	<0.001	1.0 (—)	<0.001	1.0 (—)	<0.001^†^
Yes (2,693)	59 (2.2)	2,634 (97.8)		0.42 (0.32–0.56)		0.45 (0.35–0.60)	
**Source of referral to HIV testing^§^**							
Self-referral to drop-in sites (309)	11 (3.6)	298 (96.4)	0.004	1.0 (—)	—	1.0 (—)	—
Referral by friend (1,146)	64 (5.6)	1,082 (94.4)		1.60 (0.83–3.08)	0.16	1.71 (0.89–3.29)	0.11
New social media (Wechat, Micro-blog) (1,146)	29 (2.5)	1,117 (97.5)		0.70 (0.35–1.42)	0.33	0.82 (0.40–1.66)	0.58
Outreach/Traditional media (website) (385)	12 (3.1)	373 (96.9)		0.87 (0.38–2.00)	0.75	1.16 (0.34–4.00)	0.81
Blued (10,836)	515 (4.8)	10,321 (95.2)		1.35 (0.74–2.48)	0.33	1.56 (0.84–2.87)	0.16
Others (unknown source) (2,110)	92 (4.4)	2,018 (95.6)		1.23 (0.65–2.33)	0.52	1.42 (0.75–2.71)	0.29
**First time receiving HIV testing**							
No (8,793)	357 (4.1)	8,436 (95.9)	0.001	1.0 (—)	0.001	1.0 (—)	0.007^†^
Yes (7,139)	366 (5.1)	6,773 (94.9)		1.28 (1.10–1.48)		1.32 (1.12–1.54)	
**Testing year**							
2015 (2,938)	137 (4.7)	2,801 (95.3)	0.86^¶^	1.0 (—)	—	1.0 (—)	—
2016 (6,075)	302 (5.0)	5,773 (95.0)		1.07 (0.87–1.31)	0.52	0.91 (0.73–1.14)	0.42
2017 (6,919)	284 (4.1)	6,635 (95.9)		0.87 (0.71–1.08)	0.21	0.77 (0.61–0.97)	0.03^†^
**Testing sites, location/operational dates**							
Site A (355), Downtown East/Feb 2013–Aug 2016	11 (3.1)	344 (96.9)	0.01	1.0 (—)	—	1.0 (—)	—
Site B (3,496), North West/Apr 2014–ongoing	154 (4.4)	3,342 (95.6)		1.44 (0.77–2.68)	0.25	1.25 (0.40–3.91)	0.70
Site C (547), North East/May 2017–ongoing	15 (2.7)	532 (97.3)		0.88 (0.40–1.94)	0.75	0.86 (0.25–2.98)	0.81
Site D (245), South West/May 2017–ongoing	13 (5.3)	232 (94.7)		1.75 (0.77–3.98)	0.18	1.59 (0.45–5.65)	0.47
Site E (4,313), Downtown West/Aug 2015–ongoing	173 (4.0)	4,140 (96.0)		1.31 (0.70–2.42)	0.40	1.22 (0.39–3.80)	0.74
Site F (6,975), South East/July 2012–ongoing	357 (5.1)	6,618 (94.9)		1.69 (0.92–3.10)	0.09	1.47 (0.47–4.55)	0.51

**Abbreviations:** aOR, adjusted odds ratio; CI = confidence interval; IQR = interquartile range; OR = odds ratio.

* A Beijing-based media company that operates an online dating application popular among Chinese men who have sex with men.

^†^ Statistically significant.

^§^ Percentages for the source of HIV testing information for all study participants are Blued (68%), friend referral (7.2%), WeChat/Microblog (7.2%), outreach/Danlan website (2.4%), self-admission (1.9%), and other (13.2%). WeChat is equivalent to WhatsApp in China and is operated by Tencent (https://www.tencent.com/en-us/system.html); Microblog is equivalent to Twitter in China and is operated by SINA Corp (http://english.sina.com).

^¶^ Cochran-Armitage trend test.

Overall, 723 (4.5%) of the 15,932 persons who obtained HIV testing at Blued sites during 2015–2017 had results positive for HIV. Compared with other referral sources, Blued contributed the largest proportion (71.2%, N = 515) of participants receiving HIV-positive results. The HIV-positivity rate was higher among participants aged >35 years (7.0%) than among those aged ≤35 years (4.2%) (p<0.001), among those who reported that they were not college students (5.0%) than among college students (2.2%) (p<0.001), and among those who were first-time testers (5.1%) than among repeat testers (4.1%) (p = 0.001). Participants referred from Blued for HIV testing had the second highest HIV-positivity rate (4.8%) compared with participants who were referred by a friend (5.6%). In multivariate analysis, age >35 years was associated with an increased odds of receiving an HIV-positive result (adjusted odds ratio [aOR] = 1.54; 95% confidence interval [CI] = 1.26–1.88; p<0.001), as were first-time testers, compared with repeat testers (aOR = 1.32; 95% CI = 1.12–1.54; p = 0.007). In contrast, college students were less likely to receive a positive HIV test result than were non-college students (aOR = 0.45; 95% CI = 0.35–0.60; p<0.001). An HIV-positive test result was not associated with the source of referral for HIV testing or the location at which a participant received HIV testing.

## Discussion

HIV testing volume among MSM in Beijing increased significantly at six drop-in testing sites after an online promotion campaign was deployed by Blued, the social media platform popularly used by MSM in China. These results are consistent with a previous study indicating that combining geosocial networking platforms and advertisements for HIV testing services can be an effective strategy to increase the number of MSM who obtain HIV testing (*7*). The Blued campaign was particularly effective in attracting young MSM, the population in China most affected by HIV infection (*8*).

In this analysis, first-time testers had a higher likelihood of receiving a positive HIV test result. The lower likelihood of a positive HIV test result among repeat testers might reflect a lower rate of engaging in HIV risk behaviors, the longer risk exposure among the first-time testers (lifetime) compared with that of repeat testers (time to the last negative test), or both. In 2017, an article in the China Daily reported that young college students were facing rapidly increasing risks for HIV infection (*9*). In this analysis, the rate of HIV positivity was lower among college students than among non-college students; although the majority of HIV positive test results were among MSM aged ≤35 years, only 10% of those were college students. Although the likelihood of receiving a positive HIV test result was significantly higher among participants aged >35 years, MSM in this age group accounted for <12% of the entire study population, limiting the generalizability of this finding. Other Internet-based HIV intervention projects have also had success in attracting young MSM who regularly use cellular phones for interactions with others (*10*).

The findings in this report are subject to at least three limitations. First, the data were collected as part of a programmatic activity; thus, the participants cannot be considered representative of the MSM population in Beijing. Second, college-student status was self-reported, so misclassification was possible. Finally, the project was not specifically designed to evaluate the risks for HIV infection; therefore, information that might influence HIV infection risk, including preexposure prophylaxis accessibility, condom use, and alcohol and illicit drug use, were not routinely collected.

Prioritizing the strengthening of technical assistance through partnerships with organizations actively engaging with the target population might expand the scope and reach of geosocial networking applications and facilitate understanding of users’ health behaviors, HIV testing history, and other factors that affect HIV acquisition. Further studies are needed to understand the long-term benefits of push messaging and whether it retains a detectable impact after repeated use. Optimizing the efficiency of geosocial networking applications to achieve broader testing coverage among MSM could help expand the reach of these applications in this population.

**Summary**

**What is already known about this topic?**

Men who have sex with men (MSM) are at higher risk for acquiring human immunodeficiency virus (HIV) infection and are a difficult subgroup to reach through traditional health care activities.

**What is added by this report?**

A geolocation-based online HIV testing promotion campaign from China’s largest social media platform oriented to MSM coincided with a steep continuous increase in HIV testing, suggesting the campaign is effective in promoting HIV testing among MSM.

**What are the implications for public health practice?**

Leveraging social media platforms and their geolocation-based text messaging functionality might be useful in increasing HIV testing among MSM, particularly those aged ≤35 years.

## Footnotes

* These authors contributed equally to the report.

† The ability to send a message that appears on the screen of a user of the application, even if the user is not currently logged in to the application.
